# Moral Revulsion in the Treatment of Insanity

**Published:** 1842-04-01

**Authors:** 


### MORAL REVULSION IN THE TREATMENT OF INSANITY. By *F. Leuret*, &c. &c. Paris. 1841. (Preceded by the Report made to the Royal Academy of Medicine by MM. Louis, Pariset and Double. The Report was read at the Sitting of June 1st, 1841.)

M. LEURET's work consists, on the one hand, of some general remarks on the moral treatment of insanity, especially on the value and importance of moral *diversions* in combating mental alienation. It presents, on the other hand, two striking and memorable instances of tho advantages of M. Leuret's method of treatment, which consists in leading away or *diverting* the mind from the morbid track of thoughts and associations which it has been too long pursuing.

The moral means suited to correct the aberrations of reason M. Leuret divides into two series. The first series of these moral means consists in producing a well-arranged and judicious diversion on one or more of the intellectual faculties which have still remained unaffected and intact, by giving to these faculties an unusual activity, which may absorb all the rest, and arrest seriously and uninterruptedly the undivided attention of the patient. It is a moral principle, a point which no one will dispute, that we may more easily obtain the mastery over strange and extreme mental associations by a judicious and adroit method of diversion, than by attacking them front to front, and combating them directly. This principle M. Leuret has applied to the treatment of mental alienation. The advantages he has succeeded in obtaining from this moral generalship we shall see presently.

The second series of moral means has for its object to restore the morbidly changed faculties to their normal state by acting directly on these faculties. These means consist chiefly in admonition, exhortation and advice, as also in a certain degree of authority exercised with more or less determination according to circumstances. M. Leuret has been for this latter part of his system charged with employing something like *intimidation*—such a charge however is totally groundless. It is quite clear that, under certain circumstances and towards certain characters, a determined method becomes indispensable, and is attended with great practical advantage. Between individuals whose reason is lost, and those whose reason is not yet developed; between insane persons and children there are several points of analogy; it would be no more advisable to adopt one exclusive system of moral treatment towards all insane persons, than to pursue a uniform system of education for all children. Mildness is no doubt, as the general rule, the plan to be pursued; firmness however, though the exception, often becomes necessary.

These two modes of treatment, viz. the direct and the indirect, M. Leuret employs simultaneously or successively in the same individual; sometimes he selects one to the exclusion of the other. In this particular he is determined by the character of the patient and the nature of his mental disorder. Whatever be the mode of treatment adopted, whether a direct action on the perverted functions, or a diversion on the normal functions be decided on, great patience and perseverance become necessary. One very important precept which the author inculcates is, to profit by the amendments already gained, in order to obtain new ones.

With respect to the character of the patient and its influence in deciding the plan of treatment, it is well known that a moral method or mean which would act on one patient in one way, would act on another in an entirely opposite way. One patient is sensible to kindness, and affectionate attentions; another to flattery; another again is susceptible of fear; each must be taken on his weak side.

Of those insane individuals, whose mental disorder is exempt from all complication dependent on an appreciable lesion of the encephalon, and more especially of monomaniacs, he observes that they, for the most part, enjoy good physical health, and stand in no need of pharmaceutical medication, the moral treatment alone being suited to their case. To subject such persons to blood-letting, purgatives, blisters, moxas and narcotics, &c. would be altogether unavailing and worse than unavailing. The treatment suiting their case is a moral medication; and the only agents to which we can have recourse are the mental operations, and ideas.

Before proceeding farther, we must take the liberty of expressing our objection to a very prominent term adopted by our author in this Essay, viz. the term "revulsion"—the analogy contemplated by M. Leuret, and which suggested the term, will, on more minute investigation, be found not to hold good. The term "*diversion*" is much more reconcileable to our modes of thinking.

The author avows the plan he adopted in many cases in the following words; we quote the passage, because we think it interesting and of practical application. "Around certain patients," says he, "who had been for a long time unsociable and even stupid, I contrived to collect everything calculated to awaken attention, and to excite desire in them; for want of any other means, I satisfied the greedy appetite of some for food, I created wants in others in order to stimulate their desire of correspondence with the world without; to some I allowed the indulgence of sights; to many of them music; to all, and as frequently as I could, intellectual relations with rational and reasoning individuals. Such," says the author, "is the advice I gave, and such the method I employed."

Nay, he did more; he, on some occasions, put himself face to face with the insane patient in order to struggle against him. The patient had passions; so had he; the patient had inveterate, determined passions, to which his very life seemed to cling; M. Leuret was actuated by feelings diametrically opposed to his, and he combatted him. Convinced of the truth of M. Esquirol's maxim, that, in order to be useful to the insane, one must love them, and devote himself for them, he hesitated not to engage in a struggle, the distress of which he well knew.

In the moral treatment of insanity M. Leuret considers *revulsion* as the most effectual means; its employment, however, requires precautions, without the observance of which it were vain to have recourse to it. Among these precautions the most important is to let the patient remain ignorant of the end to be attained. The reason of this is sufficiently obvious.

The two cases given by M. Leuret in illustration of his method of treatment, we shall now present, as they serve as a practical resumé of the precepts regarding moral diversion.

The first case was that of a lady about 28 years of age, who had always enjoyed good bodily health, but who had been annoyed several times before her marriage with strange fancies. Having been present one day when a female friend of hers, who was becoming a nun, was taking the veil, she fancied that it was herself who made the vows. She communicated her thoughts to her mother, who did everything in her power to convince her of her error. At a subsequent period, being present at the marriage of another female friend of hers, she took it into her head, because she had affixed her signature to the contract, that she herself was the person engaged in the contract. She communicated her ideas on this occasion also to her mother, who tried every expedient to set her right on the matter. Some time after she married, and, during the temporary absence of her husband, having returned to her mother's house, she was again annoyed by strange and absurd scruples. She fancied herself first a nun, then a priest, and ultimately a pope. Her mother, being a woman of a weak mind, did not combat these absurd notions with sufficient firmness; so that at length her imagination became so much persecuted by these absurd fancies, that she several times contemplated suicide in order to free herself from them. We must not forget to add that the lady experienced occasionally *nervous crises;* she struggled, cried, and conducted herself in such a manner as to alarm all the persons around her; she complained also of want of sleep and constant head-ache. The menses were quite regular, but at the period of their appearance there was an almost constant exacerbation of her mental delusion.

In this state of things M. Leuret undertook the management of the case. The patient had been told that he, and no one else, could cure her, and she believed it. He availed himself of this circumstance, and exacted from her a solemn promise that she would comply strictly with all his directions. He ordered her to be removed from her mother's residence, and to be removed to the house of a family who were entire strangers to her; he directed also that she should never speak of her ailments, either to any of the members of this family, nor to himself, and also that she should take such lessons as he wished her to take, either from some other person, or from himself. To this he added the use of baths, bread pills, and a ptisan rather unpleasant to the taste.

During the first month he was obliged to give all the lessons himself, as she was not disposed to pay sufficient attention to the other teachers to profit by their instructions. He gave her lessons in arithmetic, history and geography. All these restraints to which she was subjected, annoyed and thwarted her exceedingly; but as she was desirous of being cured, she submitted rather patiently. She gradually became attentive; she read, and studied with interest, and even with some degree of pleasure; she frequented different spectacles, and indulged of her own accord in all the habits of social life. At the same time her sleep returned, the pain of head had disappeared, and of her own accord she several times expressed her astonishment at having allowed herself to be carried away for so long a time by such absurd fancies.

She had now been four months under treatment, and her convalescence seemed fast approaching, though her medical attendant still dreaded a storm at each menstrual period. She then applied less to her studies, indulged more in fancies, and evinced some threatening of crises. M. Leuret however was satisfied by the previous history of the case, and especially by her conduct with respect to her mother, that her crises and mental inquietude might disappear, if she was willing to give up her indulgence in them. His efforts then were so directed as to produce this willingness in her. One day that she appeared to enjoy perfect freedom of mind and to be quite happy, she took advantage of an hour when all eyes were removed from her, to write a very desponding letter to her mother. This letter was sent to M. Leuret. Towards the period when her mental disturbance was commencing, he went to pay her a visit, and questioned her on the state of her mind, and more especially on her memory. She gave a satisfactory answer with respect to her state of mind. He then requested her to tell him what she had been engaged in doing on the preceding day, and on several preceding days, and she did so. When questioned regarding the day on which she had written the letter, she dwelt with particular pleasure on the happiness of that day, but said not a word of her having written anything. M. Leuret however drew the letter from his pocket, rebuked her rather severely, accused her of breaking her promise to him, and detailed to her the long catalogue of the symptoms of her disease, and imputed them to her as so many faults of which she had been guilty towards those whom she should have loved most. She was confounded, could only stammer out a few excuses, and he withdrew. This was the termination of her disorder—her mental inquietude did not return; the menstrual period passed off without any unpleasant symptom, and her convalescence was perfectly established.

The treatment of this case lasted for six months, during all which time the patient took no medicine whatever; she was allowed perfect liberty; M. Leuret exercising no other constraint on her, save that of allowing her to think that he would abandon her, if she did not comply with his directions to the very letter.

Here we see the first efforts of the physician were to interdict all discussion on the subject of her delirium, and to accomplish this end, he diverted her mind as intensely and as perseveringly as he could, by compelling her to fix her attention on serious and useful matters. This dependence on her physician, which was entirely voluntary on the part of the patient, lasted just as long as the disease retained any serious symptoms; according as her intellectual powers began to return, the lady evinced a certain spirit of insubordination, which prompted her to neglect her studies in order to read novels, indulge in walking abroad and in being present at various spectacles, all which enjoyments she was totally averse to before.

This case fully proves the correctness of Locke's remark that violent associations of ideas are frequently the origin of insanity.

In treating the above case M. Leuret states that he was very much assisted by the unlimited confidence which his patient had in him. In the next case however the very reverse took place. It was nothing but the dread of the remedies and the strangeness of his deportment as a physician that effected a salutary revulsion.

A lady, 35 years of age, was seized with a species of ambitious monomania; she had enjoyed perfect health both of body and mind previously; the predominant features of her delusion were of an ambitious character; such at least was the account M. Leuret obtained from the patient's mother. She was placed in a maison de santé, where, after her mental disturbance had somewhat abated, she became dumb, or rather, she ceased to speak. She understood perfectly every thing said to her, and answered in writing with precision and correctness whatever was asked her; when questioned as to the cause of her silence, she wrote that she was completely deprived of the faculty of speech by an affection of the throat. In other respects, her conduct was not altogether rational; for having no means of supporting herself, except by giving instruction, and declining to do this, she attended to no employment whatever, relying on the groundless expectation of obtaining a pension from the French government for her father's services.

Her dumbness had now lasted for eighteen months; and during all this time she had only uttered some few words in a low voice, and by way of trying; she had spoken to no person during all that time, and M. Leuret was apprized that, if he put questions to her, she would answer him readily, but in writing. M. Leuret, not wishing to have recourse to this method, was very much perplexed as to how he should proceed with her: when it occurred to him to affect dumbness with her.

This plan being determined on, he apprized the persons around her of it, impressing on them the necessity of keeping his secret; he then ordered the patient to be brought to him. He received her very coldly; placed a chair for her before the light; she sat down, and he made a sign to her to open her mouth very wide. She did so; he then pressed down the tongue, carefully inspected her throat, felt her neck, examined the state of the circulation; and when he had finished, he made a sign to her to arise and leave the room. His manner towards her was somewhat rough and abrupt, with the view of disposing her to get well as soon as possible, if it was only to get rid of his visits. His prescription was; a large plaster to be applied to the neck; mel rosæ to be applied to the bottom of the throat twenty times a day; and infusion of rhubarb, and mustard baths to the feet.

M. Leuret was much more successful than he had anticipated; as the remedies acted even before they were administered. The patient accompanied her mother to the apothecary, she saw all the preparations for the treatment to which she was about to be subjected, and, when every thing was ready, she came to her mother, and, reading with a loud voice out of some book, she uttered the following words, "I drink well, eat well, sleep well, consequently I am not sick, and have no occasion for a physician." And since that time, though it is now more than eight months since, her dumbness never returned.

With respect to this lady, as well as in the case of the other, who fancied herself pope, the appearance of the delirium was not occasioned by any appreciable physical cause, nor was it kept up by any bodily ailment. The indication to be fulfilled then was decided and positive: moral means were called for. When the brain, however, is diseased, when it is altered in its texture, and in its physical properties, the question arises, should the mental delusion dependent on this cause be also treated by moral means? M. Leuret thinks, the delusion should; but the bodily symptoms then existing, not. Physical treatment is necessary for the bodily symptoms: the moral treatment alone in such cases might be useless, or even worse than useless.

Another question presents itself; when mental alienation supervenes on the lesion of some other organ besides the brain, what is to be done? First, we must distinguish; for the two following cases may present themselves: either a sympathetic re-action is set up between the suffering organ and the brain, and there is then developed a mental aberration analogous to that observed in the acute delirium which accompanies abdominal disease, for instance: or else, the attention being directed to a state of habitual indisposition, or of pain seated in some part of the body, the individual who complains of it, gives an erroneous and incorrect explanation of it; as in the case of persons, who, when they suffer pain in the stomach, say that they are poisoned. In cases of sympathetic delusion, to ascend to the cause of the evil in order to destroy it, is undoubtedly the best and almost the only indication; in such cases physical remedies are to be employed. But in the case where to real suffering there is joined a delusive idea, or delirious conception, adopted for the purpose of explaining its nature or its cause, to meet the real suffering physical remedies are necessary; whilst, again, for the erroneous conception moral means are required.

Of the two methods employed in the moral treatment of insanity, that which acts directly against the erroneous ideas can calculate on but little success, it is less frequently applicable than the other, and is not always exempt from inconvenience. A disputation entered into with an insane person on the subject of his insanity, if it is not useful, is almost of necessity injurious, and in order to be useful, it is not only necessary that the physician should have reason on his side, but he must also make the patient feel so: otherwise the latter finds in the success of a first resistance an accession of strength which increases his conviction and obstinacy.

The same cannot, however, be said of revulsion; it never does harm, and it is most frequently serviceable. To engage an insane person with objects foreign to his insanity, is, in fact, to free him, at least temporarily, from this disease, and to impart new vigour to his understanding. The means of accomplishing this kind of revulsion are varied ad infinitum; it includes every thing which can strike the attention, awaken the imagination, or exercise the judgment; every thing, in fact, which can excite desires or passions. The skill of the physician does not then consist in discovering them, but in applying them judiciously.

For the purpose of diverting the minds of the insane, travelling, amusements, music, and, above all, bodily exertion, are recommended to them. If they are likely to prove dangerous either to themselves or to others, they are removed to special establishments, where such means of diversion as may be compatible with their state, are to be procured for them. In this particular, however, matters are but indifferently managed in these establishments. To effect real, bona fide diversion on the mind of an individual who is a prey to certain fixed ideas, to force such a person to see, feel an interest in, and busy himself with, external objects, particular attention, vigilant direction, and indefatigable surveillance, are imperatively required. Too often, however, the victims of mental alienation are suffered to pine and languish in continual torpor. Bodily labour, so profitably employed in all countries in the treatment in insanity, has already produced immense advantages; these, however, are not sufficient; for bodily labour, though favourable to the re-establishment of the intelligence, does not rectify the ideas, as intellectual labour would do. Persons may work with their hands, and go on raving at the same time for whole years, inasmuch as the work of the hands opposes but imperfectly the wanderings of thought. It is not so with intellectual toil, which concentrates the attention upon one determinate object, and compels the mind to turn away from its own morbid fancies and whimsical aberrations, and to confine itself to subjects of serious and important reality.

M. Leuret recommends that, in all retreats where insane persons are kept, schools should be established for instructing the patients, or rather the pupils, in the different branches of literature, as reading, writing, arithmetic, geography, history, &c. The establishment of such schools he considers likely to be followed by the happiest results; their effect would, he thinks, be to bring about a salutary diversion in the minds of the inmates, to promote their chances of cure, and, at all events, to render their life less melancholy than it has hitherto been.

